# Are Stem Cells Derived from Synovium and Fat Pad Able to Treat Induced Knee Osteoarthritis in Rats?

**DOI:** 10.1155/2020/9610261

**Published:** 2020-07-13

**Authors:** Reza Zare, Nader Tanideh, Behrooz Nikahval, Maryam Sadat Mirtalebi, Nasrollah Ahmadi, Shahrokh Zarea, Omid Koohi Hosseinabadi, Rohan Bhimani, Soheil Ashkani-Esfahani

**Affiliations:** ^1^Department of Veterinary Surgery, Shiraz University, Shiraz, Iran; ^2^Stem Cells Technology Research Center, Shiraz University of Medical Sciences, Shiraz, Iran; ^3^Pharmacology Department, Shiraz University of Medical Sciences, Shiraz, Iran; ^4^Department of Molecular Pathology and Cytogenetic, Shiraz University of Medical Sciences, Shiraz, Iran; ^5^Department of Veterinary Pathology, Shiraz University, Shiraz, Iran; ^6^Department of Orthopaedic Surgery, Massachusetts General Hospital, Harvard Medical School, Boston, Massachusetts, USA

## Abstract

**Background:**

Osteoarthritis (OA) is a chronic disease and a significant cause of joint pain, tenderness, and limitation of motion. At present, no specific treatment is available, and mesenchymal stem cells (MSCs) have shown promising potentials in this regard. Herein, we aimed to evaluate the repairing potentials of stem cells derived from the synovium and fat pad in the treatment of OA.

**Methods:**

Twenty-eight male rats (220 ± 20 g, aged 10-12 weeks), were randomly divided into four groups (*n* = 7): C1: nontreated group, C2: Hyalgan-treated group, E1: adipose tissue-derived stem cell-treated group, and E2: synovial membrane-based stem cell-treated group. Collagenase type II was injected into the left knee; after eight weeks, OA was developed. Then, stem cells were injected, and rats were followed for three months. Afterward, specimens and radiological images were investigated. *p* value ≤ 0.05 was set as statistically significant.

**Results:**

Compared to the C1 group, the E1 and E2 groups showed significantly better results in all six pathological criteria as well as joint space width and osteophytes of medial tibial, medial femoral, and medial fabellar condyles (*p* ≤ 0.001). Similarly, compared to the C2 group, the E1 and E2 groups had better scores regarding surface, matrix, cell distribution, and cell population viability (*p* < 0.05). E2 showed considerably higher scores compared to C2 regarding subchondral bone and cartilage mineralization (*p* < 0.05). The joint space width was similar between the C2 and E groups.

**Conclusion:**

Treatment of OA with MSCs, particularly synovial membrane-derived stem cells, not only prevented but also healed OA of the knee to some extent in comparison to the Hyalgan and nontreatment groups.

## 1. Introduction

Osteoarthritis (OA) is a common joint disorder and one of the leading causes of pain and disability, particularly in the elderly [1]. The epidemiology of this disorder is complex and multifactorial [2]. It is estimated that about 60% of the seniors up to the age of 65 years old present symptoms of OA [3, 4]. The precise pathologic mechanisms leading to the destruction of articular cartilage are unclear. Inadequate mobility may limit sufficient nourishment of the joint through synovial fluid and may lead to cell death [5, 6]. Mediators, such as catabolic cytokines and nitric oxide (NO), interleukin 1 (IL1), and tumor necrosis factor-alpha (TNF-a), modulate the production of destructive enzymes as well as the synthesis of collagen bundles and proteoglycans; these play important roles in the destruction of cartilage structures [5, 6]. Treatment methods of OA may include pharmacological therapy (i.e., intra-articular therapy, corticosteroids, capsaicin, duloxetine, topical NSAIDs, and herbal medicines), physiotherapy, surgical procedures, and tissue engineering methods [7].

Mesenchymal stem cells (MSCs) have shown noticeable potential as therapeutic agents facilitating the regeneration of the cartilaginous tissue in OA considering their multilineage potential, limited immunogenicity, and relative simplicity of growth in culture [8]. Using MSCs as an autologous source of stem cells eliminates the concerns regarding the chances of rejection and disease transmission via MSC transplant, and they are less tumorigenic compared to embryonic stem cells [9]. MSCs, in order to be able to reconstruct, refine themselves and differentiate along a mesodermal lineage, and rely on osteoblasts and chondrocytes because of their intrinsic potential in tissue repair and regeneration [10–13].

In this study, we aimed to determine and compare the efficacy of treatment of OA using MSCs derived from two different origins, synovial layer, and adipose tissue isolated from the inguinal fat pad, in rats based on pathological and radiological criteria.

## 2. Materials and Methods

### 2.1. Isolation of Mesenchymal Stem Cells

The synovial membrane-derived stem cells were harvested from the cartilage tissue of the rats' knees. Isolation of adipose tissue-derived MSCs from the inguinal fat pad was done based on a previously described method by Bunnel et al. with some alterations [14]. Characterization of the stem cells was based on flow cytometry methods [14, 15]. To prepare the stem cells, after repeated washing (3 times here) of the acquired specimen with a solution containing phosphate-buffered saline and supplemented with 1% penicillin-streptomycin (PBS-PS; Sigma, USA) until all connective tissues and blood vessels were liberated, the sample was minced into smaller parts. The specimen was then digested using 0.1% collagenase type I 37 ± 1°C and shaking for 3 hours. Then, an equal volume of Dulbecco's modified Eagle's medium (DMEM; Biovet, Bulgaria) containing 10% fetal bovine serum (FBS; Biovet, Bulgaria) was added to the mixture. The suspension was then filtered through a 100 *μ*m filter (Falcon, USA) to remove the redundant solid aggregates and was centrifuged at 1500 rpm for 5 minutes at a temperature of 24 ± 2°C. Using 1 mL of lysis buffer (Promega, Germany), the pellet was resuspended, incubated (15 minutes), washed using PBS-PS, and centrifuged at 1500 rpm for 5 minutes, in order to clear the suspension from red blood cells. The supernatant was then removed, and the pellet was resuspended in a complete medium containing DMEM, 20% FBS, and 1 penicillin-streptomycin in 75 cm^2^ culture flask and held in an incubator with a humidified atmosphere of 37°C and 5% CO_2_.

### 2.2. Induction of Osteoarthritis and Grouping

The study protocol was approved by the medical ethics committee of Shiraz University of Medical Science, Shiraz, Iran (Reg. no. 97-01-67-19128). OA was induced by using the intra-articular injection of collagenase type II. At first, the rats were anesthetized by using a mixture of Ketamine (10% Alfasan, Netherlands, 100 mg/kg for rat) and Xylazine (2% Rompun Bayer, Germany, 10 mg/kg for rat); then, in aseptic surgery condition, collagenase type II was injected into the left knees of the rats, and after 8 weeks, OA was developed. Treatments were started after OA was developed (8 weeks after injection of collagenase type II) [16].

Twenty-eight *Sprague Dawley* male adult rats, weighing 220 ± 20 g and aged between 10 and 12 weeks, were randomly divided into 4 groups (*n* = 7) as follows: C1—nontreated control group, rats did not receive any treatment; C2—Hyalgan-treated control group, rats received 0.1 cc intra-articular Hyalgan (Fidia, Italy) once every two weeks during the treatment as described by Jo et al. [17]; E1—stem cell-treated group 1, rats were injected intra-articular adipose tissue-derived stem cells with a dose of 2.5 × 10^6^ in a volume of 50 *μ*L once during the treatment; E2—stem cell-treated group 2, rats were injected with synovial membrane-based stem cells with a dose of 2.5 × 10^6^ cells in a volume of 50 *μ*L once during the treatment.

### 2.3. Radiological and Pathological Evaluations

After the treatments, the rats were followed up for three months; then, radiological imaging was done in anterior-posterior (AP) and lateral positions. All images were taken by the same operator and equipment (Axiom Multix M radiographic unit, Siemens™, Germany) and using a standard method. Grading was done by a blind observer by using the International Cartilage Repair Society (ICRS) scores (Table 1).

Rats were euthanized with CO_2_ 70% at the end of the third month. Specimens from the knee joint were obtained and fixed in 10% buffered formaldehyde and then were transferred into paraffin. Serial sagittal sections were provided and stained with hematoxylin and eosin (H&E, for cellular architecture), Toluidine blue, and Safranin O (for proteoglycan contents). All pathological specimens were assessed by a pathologist who was blinded from the study data. The degree of cartilage repair of each rat was evaluated based on ICRS scores which consisted of 6 indices including surface, matrix, cell distribution, cell population viability, subchondral bone, and cartilage mineralization (Table 2).

### 2.4. Statistical Analysis

All nonparametric data were analyzed by using Kruskal-Wallis and Mann–Whitney *U* tests through SPSS software 21.0 (IBM, USA). Data are presented as the median ± interquartile range (IQR), and the significance level of the *p* value was less than 0.05.

## 3. Results

**Figure 1:**
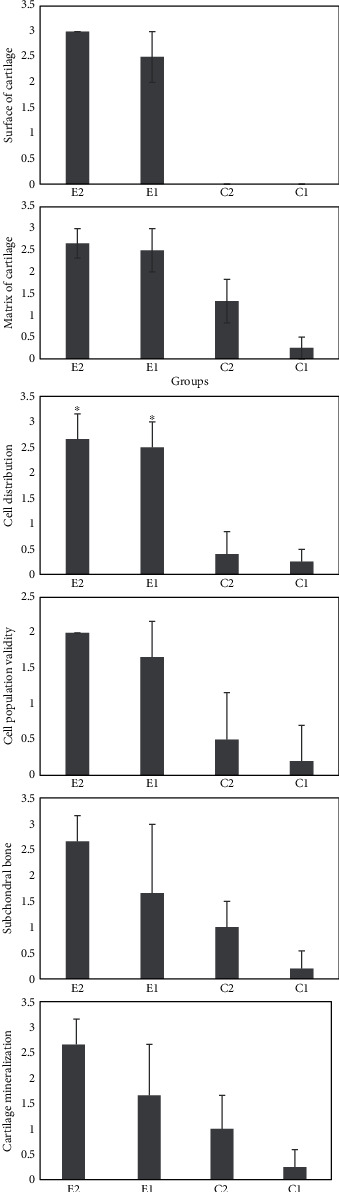
Histological scores of OA in rat models including E2 group: synovial membrane-based stem cell, E1 group: adipose tissue-derived stem cells, C2 group: Hyalgan-treated, and C1 group: nontreated group, based on International Cartilage Repair Society (ICRS). Values are shown as the median and interquartile range. ^∗^*p* value < 0.05 vs. C1.

**Figure 2:**
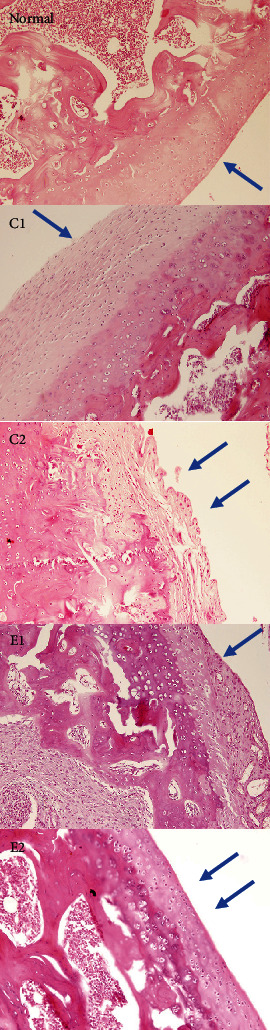
A typical histological section from all groups' surface of articular cartilage (H&E, ×200). E2 group: synovial membrane-based stem cells, arranged chondrocyte in columnar cluster along with continuous and smooth surface of articular hyaline cartilage with no foci of abnormal calcification; E1 group: adipose tissue-derived stem cells, the surface of articular cartilage was mainly continues composed of fibrocartilage tissue arranged in clusters, and the subchondral bone was detached with increased remodeling; C2 group: treated with Hyalgan, the surface of articular cartilage was continuous mainly composed of fibrocartilage with foci of hyaline cartilage cluster; and C1 nontreated group, the surface of articular cartilage was completely destructive and composed of disorganized fibrocartilage and fibrous tissues, and the subchondral bone was detached.

Pathologic evaluations showed that both stem cell-treated groups, E1 and E2, had significantly higher scores concerning cartilaginous surface, matrix, cell distribution, viability, subchondral bone, and cartilage mineralization, in comparison with the C1 group (*p* < 0.001, Figure 1). The C2 group showed significantly higher scores concerning the matrix (*p* < 0.001), cell viability (*p* = 0.03), subchondral bone (*p* = 0.001), and cartilage mineralization (*p* = 0.004), compared to the C1 group. Stem cell-treated groups had better results regarding surface (*p* < 0.001), matrix (*p* < 0.001 and *p* = 0.01, respectively), cell distribution (*p* = 0.009 and *p* = 0.011, respectively), and cell population viability (*p* = 0.016 and *p* = 0.021, respectively) in contrast with the Hyalgan-treated group—C2; however, considering subchondral bone and cartilage mineralization, only E2 showed considerably higher scores compared to C2 (*p* = 0.021 and *p* = 0.013, respectively) (Figures 1 and 2).

**Figure 3:**
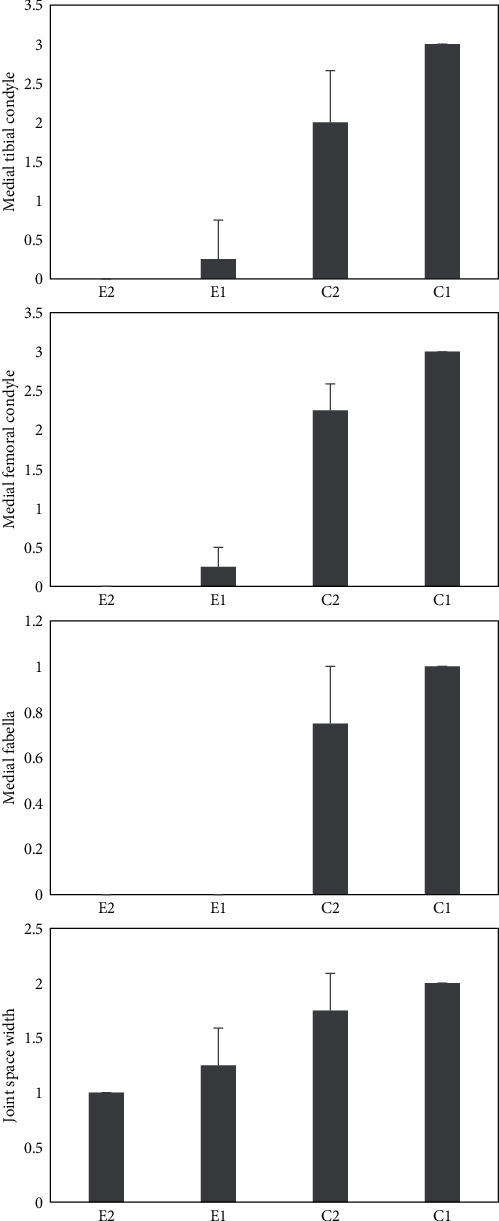
Radiological scores regarding OA in rat models including E2 group: synovial membrane-based stem cells, E1 group: adipose tissue-derived stem cells, C2 group: treated with Hyalgan, and C1 group: nontreated, based on International Cartilage Repair Society (ICRS). Values are shown as the median and interquartile range. ^∗^*p* value < 0.05 vs. C1.

Radiographic images depicted that the E1 and E2 groups had significantly lower scores indicative of better healing effects regarding medial tibial condyle osteophytes (*p* < 0.001), medial femoral condyle osteophytes (*p* < 0.001), medial fabellar condyle osteophytes (*p* < 0.001), and joint space width (*p* = 0.03 and *p* = 0.02, respectively) compared to the C1 group. The C2 group showed lower scores compared to C1 concerning medial tibial condyle score (*p* = 0.04); moreover, the E1 and E2 groups showed significantly lower scores compared to C2 regarding medial tibial condyle (*p* < 0.001), medial femoral condyle (*p* < 0.001), and medial fabellar condyle (*p* < 0.001). The joint space width score was significantly lower merely in the E2 group (*p* = 0.01, respectively) (Figure 3).

## 4. Discussion

Currently, the treatment of OA is mainly focused on symptom therapies, pain reduction, and improving the quality of life [18, 19]. There is no specific treatment either for stopping the progress of OA or vanishing the underlying causes [20]. In this study, we aimed to evaluate the efficacy of the treatment of OA using MSCs derived from two various origins, adipose tissue, and synovial membrane. Another aim of this study was to compare these treatments with intra-articular Hyaluronic acid (Hyalgan) as a potential agent that was shown to alleviate the degenerative process in the joint [21]. Our results showed that both adipose tissue-derived and synovial membrane-derived stem cells possess promising effects in reducing the degeneration of the joint cartilage based on radiologic and pathologic parameters compared to the untreated control group. Moreover, compared to Hyalgan, stem cell treatment results were significantly better regarding all the evaluated parameters, although Hyalgan has also shown significant improvements in the joint regarding cartilage matrix, cell viability, subchondral bone, and cartilage mineralization compared to the untreated control group. Comparing the results of synovial and adipose-derived MSCs with the control group, regarding some parameters including subchondral bone and cartilage mineralization, synovium-derived MSCs showed even better effects on the joint affected with OA.

According to the outcome of the present study, a single intra-articular injection of 2.5 × 10^6^ adipose tissue-derived MSCs exert a protective impact on the OA in rats compared to the nontreated group. Radiologic evaluation of the joint, as well as the pathologic results comparing this treatment with Hyalgan, as a currently used treatment for OA, also depicted the superiority of this method of treatment. Adipose tissue is an easy access rich source of MSCs, and stem cells obtained from adipose tissue have remarkable proliferation and differentiation potentials into chondrocytes that can be due to secretion of various signaling molecules and growth factors by these cells [22, 23]. In a study by Toghraie et al. MSCs derived from infrapatellar fat pad were used to treat OA induced by anterior cruciate ligament (ACL) dissection in rabbits [24]. They showed that a single intra-articular injection of 10^6^ MSCs led to decreased cartilage degeneration, subchondral sclerosis, and osteophyte formation compared to the nontreated group. Kuroda et al. also observed decreased joint erosions in both lateral and medial femoral condyles, suppressed cartilage degeneration, and increased cell viability, treating OA-induced rabbits with adipose tissue-derived MSCs [25]. ter Huurne et al. reported positive outcome using the inguinal fat pad in mice model of OA induced by collagenase [26]. In their study, the MSC-treated group showed reduced synovial thickening, enthesophyte formation, and cartilage destruction compared to nontreated mice. Additionally, in human case-control studies in 2012 and 2013 by Koh et al., promising results were presented after treating patients suffering from knee OA with MSCs obtained from infrapatellar fat pad [27, 28]. After 25 intra-articular injections of MSCs combined with arthroscopic debridement in the patients, compared to the control group that had undergone arthroscopic debridement and platelet-rich plasma injections, the patients showed significant improvement in activity as well as radiologic and arthroscopic evaluation of the joint in both short- and long-term follow-ups [27, 28]. Overall, several published papers as well as the results of the present study show that adipose tissue can play the role of a noticeable source for MSCs useful in the treatment of OA. However, clinical evidence in this regard seems to be still unsatisfactory [29–33].

Our results demonstrated that a single injection of 2.5 × 10^6^ synovial MSCs into the knee joint can have a protective effect in collagenase-induced OA in rats based on pathologic and radiologic evaluation of the joint. The efficacy of this treatment was also superior to Hyalgan. The synovial membrane has an intrinsic potential of regeneration leading to its recovery after synovectomy, suggesting that it can play a considerable role as a stem cell resource in cartilage repair [34, 35]. Zhu et al. in collagenase-induced OA mouse models showed that MSC-derived exosomes which contain various modulatory factors for cellular signaling, inflammatory response, and differentiation can enhance the protective effect of synovium-derived MSCs after a single injection into the joint; however, these exosomes have shown a stronger stimulatory effect on human-induced pluripotent stem cells compared to the synovial MSCs in the OA joint [36]. Mak et al. in a surgical-induced knee joint cartilage defect in mice showed that intra-articular injection of 10^4^ synovial MSCs with a specific cell marker (Sca-1) protects the joint against the progression of the cartilage defect [37]. Neybecker et al. using human synovium-derived MSCs in rat models of OA induced by ACL dissection depicted that these cells have a noticeable capacity for chondrogenic gene induction and synthesis of the extracellular matrix, but they observed that intra-articular injection of these xenogenic MSCs obtained from human did not show significant chondroprotection in OA rat models [38]. Ozeki et al. have used synovial MSCs in OA induced via ACL dissection [15]. They, for the first time according to their claim, compared the chondroprotective effects of repeated injections of MSCs with a single injection of 10^6^ MSCs using fluorocytometric methods as well as pathologic evaluation of tibial plateau and femoral condyle. According to their outcome, the weekly injection of MSCs has superior efficacy compared to a single injection in inhibiting OA progression and maintaining the viability of MSCs. They also reported that synovial MSCs can retain their stem cell properties after migration to the synovial tissue without differentiating into another lineage. However, according to their report single injection of 10^6^ MSCs may not be beneficial since migrated MSCs do not survive in the long term and repeated injection is needed to maintain the number of MSCs in the joint. Conclusively, approved by the present study and the previously published papers, synovial MSC seem to be a promising source of stem cell in the treatment of OA; thus, the number of injected stem cells or repeating the injections aiming to maintain the number of viable cells seems to play an important role and should be further investigated.

This study had some limitations to be mentioned. First, though we used a high dose of MSCs for injection (2.5 × 10^6^) we should have considered performing also repeated injection method that could show even better results. However, according to limited facilities, that goal was not obtainable. Secondly, we were not able to estimate inflammatory markers and count the MSCs within the joint using flow cytometry to provide more reliable results in this regard. Lastly, the number of the rats in each group could be higher in order to prove more valid and more reliable outcome.

## 5. Conclusion

The treatments of OA with MSCs, derived from synovial membrane and adipose tissue, were useful and effective, in comparison to the nontreated group as well as Hyalgan-treated rats, according to the radiological and histopathological analyses. The result of this study was in accordance to the majority of previously published papers on this subject. The differences in the outcomes can be related to the method used to create the AO model, the duration of the study, and the number of injection repeats as well as the number of injected MSCs. Thus, conducting further studies with different designs and greater population as well as human trials is necessary to establish the advantages and disadvantages of stem cell therapy, specifically with the above-mentioned cell sources, in OA.

## Figures and Tables

**Table 1:** Radiological grading for knee osteoarthritis (OA) in rat models.

Radiographic OA feature of medial compartment		Grade 0	Grade 1	Grade 2	Grade 3
Joint space width		Normal	Reduced	Absent	N/A
Osteophyte	Medial tibial condyle	Absent	Small	Moderate	Sever
	Medial femoral condyle	Absent	Small	Moderate	Sever
	Medial fabella	Absent	Present	N/A	N/A
Total osteophyte		0-7			
Global OA score		0-9			

**Table 2:** Histopathological grading using international cartilage repair society (ICRS) histological score.

Variables	Scores
Surface	
Smooth/continuous	3
Discontinuous/irregular	0
Matrix	
Hyaline	3
Mixture: hyaline+fibrocartilage	2
Fibrocartilage	1
Fibrous tissue	0
Cell distribution	
Columnar	3
Mixture/columnar cluster	2
Cluster	1
Individual cell/disorganized	0
Cell population variability	
Predominantly viable	3
Partially viable	1
<10% viable	0
Subchondral bone	
Normal	3
Increase remodeling	2
Bone necrosis/granulation tissue	1
Detached/fracture/cell at base	0
Cartilage mineralization	
Normal	3
Abnormal/inappropriate location	0

## Data Availability

The experimental data of the study is available on demand.

## Abbreviations

OA: Osteoarthritis
MSCs: Mesenchymal stem cells
ICRS: International Cartilage Repair Society
IFP: Infrapatellar fat pad.
